# Surgical volume and conversion rate in laparoscopic hysterectomy: does volume matter? A multicenter retrospective cohort study

**DOI:** 10.1007/s00464-017-5780-x

**Published:** 2017-08-25

**Authors:** José H. M. Keurentjes, Justine M. Briët, Geertruida H. de Bock, Marian J. E. Mourits

**Affiliations:** 1 Department of Gynaecologic Oncology, University of Groningen, University Medical Center Groningen, Groningen, The Netherlands; 2 Department of Epidemiology, University of Groningen, University Medical Center Groningen, Groningen, The Netherlands

**Keywords:** Annual surgical volume, Laparoscopic hysterectomy, Conversion rate, Laparotomy, Surgical skills

## Abstract

**Background:**

A multicenter, retrospective, cohort study was conducted in the Netherlands. The aim was to evaluate whether surgical volume of laparoscopic hysterectomies (LHs) performed by proven skilled gynecologists had an impact on the conversion rate from laparoscopy to laparotomy.

**Methods:**

In 14 hospitals, all LHs performed by 19 proven skilled gynecologists between 2007 and 2010 were included in the analysis. Surgical volume, conversion rate and type of conversion (reactive or strategic) were retrospectively assessed. To estimate the impact of surgical volume on the conversion rate, logistic regressions were performed. These regressions were adjusted for patient’s age, Body Mass Index (BMI), ASA classification, previous abdominal surgery and the indication (malignant versus benign) for the LH.

**Results:**

During the study period, 19 proven skilled gynecologists performed a total of 1051 LHs. Forty percent of the gynecologists performed over 20 LHs per year (median 17.3, range 5.4–49.5). Conversion to laparotomy occurred in 5.0% of all LHs (53 of 1051); 38 (3.6%) were strategic and 15 (1.4%) were reactive conversions. Performing over 20 LHs per year was significantly associated with a lower overall conversion rate (OR_adjusted_ 0.43, 95% CI 0.24–0.77), a lower strategic conversion rate (OR_adjusted_ 0.32, 95% CI 0.16–0.65), but not with a lower reactive conversion rate (OR_adjusted_ 0.96, 95% CI 0.33–2.79).

**Conclusion:**

A higher annual surgical volume of LHs by proven skilled gynecologists is inversely related to the conversion rate to laparotomy, and results in a lower strategic conversion rate.

Hysterectomy is one of the most frequently performed surgical procedures in gynecology. An increase was seen in laparoscopic hysterectomies (LHs) over the last decade, mostly at the expense of the number of abdominal hysterectomies (AHs) [1, 2]. Several randomized controlled trials have shown that LH is an effective and safe alternative to AH for benign indications and early stage, low-risk endometrial cancer. A shorter hospital stay, less pain, less blood loss, quicker return to daily activities and better short-term quality of life in favor of laparoscopy have been reported [3–9].

LH is an advanced laparoscopic procedure and is known for its learning curve [10–12]. Little is known about how to maintain or improve surgical skills after the learning curve. Previously, the impact of surgical volume on surgical skills has been mentioned as a factor of importance [13–15]. In the Netherlands, an annual volume of 20 is recommended for several complex surgical procedures. The Dutch Health Care Inspectorate (IGZ) stated in 2011 that for high complex procedures a minimum of twenty treatments per year per team is required. As a laparoscopic hysterectomy is a level 3 (high complex) procedure, the same criteria are applicable [16]. However, there is scarce scientific evidence and no consensus on the annual number of advanced laparoscopic procedures per surgeon needed to maintain the skilled [15, 17]. A recent systematic review on gynecologic procedures concluded that performing a procedure once a month or less resulted in higher rates of adverse outcomes [18].

There are data that suggest that the conversion rate from laparoscopy to laparotomy when performing a LH could be used as an indicator of surgical skills [18, 19]. A conversion is defined as the need to switch from laparoscopy to laparotomy at any time during the procedure. An important distinction can be made between a *strategic* conversion to prevent an adverse event which is a decision made at the beginning of the laparoscopic surgery (<15 min); and a *reactive* conversion that often occurs as a consequence of a complication or after considerable time in the procedure [20, 21]. This study investigated the impact of annual surgical volume on the occurrence as well as the type of conversion from laparoscopy to laparotomy when performed by a proven skilled surgeon. Does a larger volume of advanced laparoscopic procedures per year, in certified competent surgeons, prevent conversions?

## Materials and methods

This study was performed following an earlier randomized controlled trial on the safety of laparoscopy versus laparotomy in early stage endometrial cancer [4, 22]. In this trial, gynecologists could only participate after completing a learning curve for LH [12]. This learning curve was completed when having passed the cut-off score of an objective structured assessment of technical skills (OSATS) twice, while performing a LH [4, 12, 22]. Of the 26 proven skilled surgeons who participated in the randomized controlled trial, 19 agreed to participate in the current study. All these surgeons were trained to perform a LH according to the same surgical protocol as described earlier [22]. From January 2007 to 2010, all LHs performed by these 19 proven skilled surgeons were retrieved using standardized health insurance codes in the hospital databases. All consecutive LH’s during the study period were included, irrespective of indication. The following data were collected from the operation records: name of surgeon(s), patient characteristics [age, body mass index (BMI), ASA classification, and previous abdominal surgery], indication for LH (malignant or benign), and if a conversion occurred. Conversions were rated as strategic or reactive. In case of a combination of reasons (e.g. adhesions and uncontrollable bleeding), the conversion was rated as reactive because of the ‘no choice’ option [20]. The reason for conversion was cross-checked independently by two authors (JHMK and JMB). Both authors agreed on type of conversion (strategic or reactive) in all cases. In case of any doubt, this was discussed with a third author (MJEM). All data were anonymized and entered into a password-protected database.

### Statistics

Patient characteristics were stratified by conversion (none, reactive or strategic). Differences between these three groups regarding descriptive statistics were tested using Chi-square tests or Kruskal–Wallis tests. In 3% of all cases, the BMI data were missing; the mean BMI was imputed for these missing values. Annual volume per surgeon was defined as the total number of LHs performed during the study period of three years, divided by three. When two participating gynecologists performed a LH together (which occurred in 42 of the 1051 procedures), the procedure counted for the volume of both gynecologists.

Univariate logistic regression analysis was performed with conversion to laparotomy as the dependent variable and annual volume for LHs (>20 versus ≤20), based on the statement of the Dutch Health Care Inspectorate (IGZ) [16]. The patient age, BMI, ASA classification (≥2 versus 1), previous abdominal surgery (yes versus no), and indication for LH (malignant versus benign) were used as independent variables.

In the multivariate logistic regression, the effect of a higher annual volume for LHs (>20 versus ≤ 20) was assessed and adjusted for all other covariates. In this way, odds ratios (ORs) and 95% confidence intervals (95% CIs) were calculated. These analyses were also done with reactive and strategic conversions as the dependent variables. Analyses were performed using the SPSS^®^ software package, version 20.0 for Windows (SPSS Inc., Chicago, Illinois, USA). The level for statistical significance was set at *p* < 0.05 for all tests.

## Results

### Patient characteristics

A consecutive series of 1051 LHs performed by 19 proven skilled gynecologists was included during the research period. Mean age of the patients was 50.6 years (SD = 12.0) and mean BMI was 27.5 kg/m^2^ (SD = 6.1). In 80.5% of the LHs (*n* = 846) there was a benign indication for the operation; and 205 LHs (19.5%) were performed in patients with an early stage, low-risk endometrial cancer (Table 1).

**Table 1:** Patient characteristics stratified by conversion from LH to laparotomy (none, reactive or strategic; *N* = 1051)

	No conversion *N* = 998 (95%)	Strategic conversion *N* = 38 (3.6%)	Reactive conversion *N* = 15 (1.4%)	*P*
Age				
Median (range)	47.0 (21.0–89.0)	56.0 (37.0–79.0)	66.0 (36.0–87.0)	<0.0001^*^
Body mass index				
Median (range)	26.2 (16.0–52.7)	29.4 (18.2–44.8)	28.6 (20.0–46.9)	<0.001^*^
ASA classification	(*n* = 982)			<0.0001
1	548 (54.9%)	12 (31.6%)	3 (20.0%)	
≥2	434 (43.5%)	26 (68.4%)	12 (80.0%)	
Previous abdominal surgery				
No	802 (80.4%)	28 (73.7%)	12 (80.0%)	0.60
Yes	196 (19.6%)	10 (26.3%)	3 (20.0%)	
Indication for LH				
Benign	816 (81.8%)	22 (57.9%)	8 (53.3%)	<0.0001
Malignant	182 (18.2%)	16 (42.1%)	7 (46.7%)	

* Tested with Kruskal–Wallis

*ASA* American Society of Anesthesiologists

### Conversions

A conversion from laparoscopy to laparotomy occurred in 53 out of 1051 (5.0%) of the LHs; 38 (3.6%) were strategic and 15 (1.4%) were reactive conversions. The main reason for a strategic conversion was an enlarged/immobile uterus (*n* = 15) or adhesions/inadequate exposure (*n* = 14, Table 2). Univariate analysis showed that a higher annual volume of LHs per surgeon was related to a lower conversion rate. An annual volume of over 20 LHs resulted in a significantly lower conversion rate (OR 0.34, 95% CI 0.19–0.59, Table 3). Eleven gynecologists (58%) performed 20 LHs or fewer per year and of the 370 LHs they performed 32 conversions occurred (8.6%). Eight gynecologists (42%) performed more than 20 LHs annually and of the 681 LHs they performed 21 (3.1%) conversions occurred. Higher age and higher BMI increased the risk for a conversion (OR 1.05, 95% CI 1.03–1.07 and OR 1.08, 95% CI 1.04–1.12, per year and BMI point, respectively). Women with an ASA classification ≥2 and women with a malignant indication for LH were also at an increased risk for a conversion (OR 3.20, 95% CI 1.74–5.89 and OR 3.44, 95% CI 1.95–6.06, respectively, Table 3).

**Table 2:** Primary reason for strategic and reactive conversions in LH (*N* = 53 out of 1051; 5%)

Conversions (*n* = 53)		*N* ^a^ (%)
Type	Indication	
Strategic (*n* = 38; 72%)	Immobile/enlarged uterus	15 (39.5%)
	Adhesions/inadequate exposure	14 (36.8%)
	Technical difficulties (too short trocar in the very obese + impossibility for Trendelenburg position)	3 (7.9%)
	Suspicion of advanced malignancy	6 (15.8%)
Reactive (*n* = 15; 28%)	Uncontrollable bleeding	8 (53%)
	Lesions (e.g. bladder or bowel lesions)	3 (20%)
	Inadequate exposure after a considerable amount of dissection	3 (20%)
	Technical difficulty (broken morcellator)	1 (7%)

^a^A conversion can be conducted because of a single or multiple reason

**Table 3:** Predictors for risk for all conversions (*n* = 53) from LH to laparotomy

	Univariate analysis		Multivariate analysis	
	OR	95% CI	OR	95% CI
Annual volume				
≤20	**1**		**1**	
>20	0.34	(0.19–0.59)	0.43	(0.24–0.77)
Age of patient^a^	1.05	(1.03–1.07)	1.02	(0.99–1.05)
BMI^a^	1.08	(1.04–1.12)	1.05	(1.00–1.09)
ASA classification				
1	1		1	
≥2	3.20	(1.74–5.89)	1.94	(0.98–3.86)
Previous abdominal surgery				
No	1		1	
Yes	1.33	(0.70–2.53)	1.61	(0.82–3.16)
Indication for LH				
Benign	1		1	
Malignant	3.44	(1.95–6.06)	1.61	(1.00–1.09)

^a^Each increase in year of age or point in BMI in the patient is associated with an increase in risk for a conversion

In the multivariate analysis, annual volume of over 20 LHs per surgeon was also significantly associated with fewer conversions (OR_adjusted_ 0.43, 95% CI 0.24–0.77, Table 3).

An annual volume of more than 20 LHs was associated with fewer strategic conversions (OR_adjusted_ 0.31, 95% CI 0.15–0.63, Table 4). The risk for a reactive conversion was not related to an annual volume of more than 20 LHs (OR_adjusted_ 0.96, 95% CI 0.33–2.82, Table 5). The only risk factor for a reactive conversion was increasing age (OR_adjusted_ 1.06, 95% CI 1.01–1.11).

**Table 4:** Predictors of risk for strategic conversions (*n* = 38) from LH to laparotomy

	Univariate analysis		Multivariate analysis	
	OR	95% CI	OR	95% CI
Annual volume				
≤20	1		1	
>20	0.27	(0.14–0.53)	0.31	(0.15–0.63)
Age of patient^a^	1.04	1.01–1.06	1.00	(0.97–1.04)
BMI^a^	1.08	1.03–1.12	1.05	(1.00–1.01)
ASA classification				
1	1		1	
≥2	2.68	1.34–5.40	1.79	(0.82–3.91)
Previous abdominal surgery				
No	1		1	
Yes	1.46	(0.70–3.06)	1.77	(0.82–3.81)
Indication for LH				
Benign	1		1	
Malignant	3.2	(1.63–6.15)	1.90	(0.80–4.51)

^a^Each increase in year of age or point in BMI of the patient is associated with an increased risk for a conversion

**Table 5:** Predictors of risk for reactive conversions (*n* = 15) from LH to laparotomy

	Univariate analysis		Multivariate analysis	
	OR	95% CI	OR	95% CI
Annual volume				
≤20	**1**		**1**	
>20	0.62	(0.22–1.71)	0.96	(0.33–2.82)
Age of patient^a^	1.07	(1.04–1.11)	1.06	(1.01–1.11)
BMI^a^	1.07	(1.00–1.15)	1.04	(0.96–1.12)
ASA classification				
1	1		1	
≥2	4.87	(1.37–17.36)	2.21	(0.53–9.20)
Previous abdominal surgery				
No	1		1	
Yes	1.01	(0.28–3.60)	1.15	(0.31–4.20)
Indication for LH				
Benign Malignant	1 3.70	(1.33–10.33)	1 1.09	(0.33–3.65)

^a^Each increase in year of age or point in BMI of the patient is associated with an increased risk for a conversion

## Discussion

In this large consecutive series of 1051 LHs performed by proven skilled gynecologists, the mean observed conversion rate was 5.0%; 38 (3.6%) of the conversions were strategic and 15 (1.4%) were reactive. Performing over 20 LHs per year was significantly associated with a lower overall conversion rate and a lower strategic conversion rate, but not with a lower reactive conversion rate.

The reported conversion rate to laparotomy during LH in different studies ranges from 0 to 19% (mean = 3.5%) [20]. The observed mean conversion rate of 5.0% in this study is on the lower end of this range which might be explained by the fact that the current study only included proven skilled gynecologists. In our multivariate analysis, former laparotomy, comorbidity and indication for surgery were not associated with the conversion rate. We found a higher conversion rate in obese and older women, in accordance with previous studies [23–25].

Several studies report on the impact of surgical volume on conversion rate in LH and some confirm our finding that higher annual surgical volume is associated with a lower conversion rate [18, 26–28]. A conversion in itself is not a complication or failure of the surgeon; on the contrary, a conversion can be a decision that warrants patients’ safety. In our cohort, we observed a lower strategic conversion rate by surgeons with higher annual volume; in other words, the ability to finish a procedure laparoscopically improves with higher volume, as was found by others [20]. The reactive conversion rate did not diminish with more procedures per year. This might imply that higher exposure does not result in an ability to prevent ‘per-operative’ problems, which was also described by others [15]. As reactive conversions are associated with higher postoperative morbidity, documentation of a conversion and its indication is important [20].

The LH procedures in this study have been performed up to 2010. In the past 6 years, the indications for laparoscopic procedures have increased. Moreover, technical possibilities and training of surgeons and surgical teams have improved. Although a laparoscopic hysterectomy has become more mainstream, there is still a discussion on quantity of annual volume and how to maintain the skilled as part of the ‘quality check’ and for patients safety. Possibly concentration of the LH, and other level 3 and 4 laparoscopic procedures, to a few surgeons per hospital could be necessary for surgeons to stay competent and to minimize conversions due to relatively little exposure. In addition, we could take into account the case mix (benign/malignant indication, patient characteristics such as obesity etc.). A recent study showed a nice example of a quality tool that could help improve performances and incorporates case mix as well [29].

Further prospective research is needed to find out what the minimum number of advanced laparoscopic procedures per surgeon per year is, after the learning curve, to result in an acceptable conversion rate and complication rate.

One of the strengths of this study is that it was performed in the aftermath of a large randomized controlled trial [4], in which all gynecologists were proven skilled according to a uniform standardized assessment tool, in performing a LH according to a standardized surgical procedure [22]. The additional distinction in reactive and strategic conversions gives insight into the reason for the conversion. One of the limitations is the retrospective study design, although conversion from laparoscopy to laparotomy is a distinct event, which is documented and recorded clearly in the patient file, the surgical report and hospital administration, and it cannot be missed nor denied.

Another limitation is that we did not take into account how many other level 3 or 4 laparoscopic procedures (other than LH) our participating gynecologists performed during the study period.

In conclusion, this large consecutive series of LHs performed by proven skilled gynecologists shows that higher annual surgical volume results in a lower strategic conversion rate to laparotomy.
